# Dynamic changes of immune cells and therapeutic responses in experimental models of COPD

**DOI:** 10.3389/fimmu.2026.1698508

**Published:** 2026-02-25

**Authors:** Jingxian Xie, Pengfei Li, Jianjun Du, Shiran Li, Zhimin Li, Jiao Zhang, Siyu Zeng, Yanqiu Zhang, Yong Yang

**Affiliations:** 1 Department of Pharmacy, Personalized Drug Research and Therapy Key Laboratory of Sichuan Province, Sichuan Provincial People’s Hospital, School of Medicine, University of Electronic Science and Technology of China, Chengdu, China; 2 School of Pharmacy, Southwest Medical University, Luzhou, Sichuan, China; 3 Department of Pharmacy, Bishan Hospital of Chongqing Medical University, Bishan Hospital of Chongqing, Chongqing, China

**Keywords:** chronic obstructive pulmonary disease, experimental models, immune cells, Chronic inflammation, targeted immunotherapy

## Abstract

Chronic obstructive pulmonary disease (COPD) is a heterogeneous respiratory disorder characterized by a complex pathogenesis involving chronic inflammation, protease–antiprotease imbalance, oxidative stress, and epigenetic regulation. Experimental models, including cigarette smoke exposure, air pollution, and acute exacerbation models, provide essential platforms for investigating immune cell dynamics during disease initiation and progression. Macrophages contribute to inflammatory amplification and tissue destruction through polarization imbalance and metabolic reprogramming. Neutrophils exacerbate persistent lung injury via recruitment, protease release, NET formation, and delayed apoptosis, while also promoting airway remodeling during the repair phase. T cells—particularly CD8^+^, Th1/Th17, and tissue-resident memory T cells—sustain chronic inflammation through cytotoxicity and cytokine networks, whereas impaired Treg function hinders inflammation resolution. Additional immune populations, such as NK cells, eosinophils, and fibrocytes, further drive inflammatory amplification and fibrotic remodeling. Therapeutic explorations targeting distinct inflammatory phenotypes indicate that conventional glucocorticoids and PDE4 inhibitors remain beneficial in eosinophil-driven inflammation, whereas biologics targeting IL-5, IL-13/IL-4, TSLP, and IL-33 have produced variable outcomes in COPD clinical trials. These findings highlight the importance of precision phenotyping and personalized immunomodulatory strategies. Overall, systematic elucidation of immune cell dynamics in COPD experimental models provides new insights into mechanisms of inflammation persistence and therapeutic responses, offering a theoretical basis for developing targeted interventions.

## 1 Introduction

Chronic Obstructive Pulmonary Disease (COPD) is a heterogeneous lung condition characterized by chronic respiratory symptoms (dyspnea, cough, sputum production and/or exacerbations) due to abnormalities of the airways (bronchitis, bronchiolitis) and/or alveoli (emphysema) that cause persistent, often progressive, airflow obstruction (1). As the third leading cause of death among non-communicable diseases, the health burden imposed by COPD continues to rise globally (1). The pathogenesis of COPD involves complex multi-faceted and multi-level interactions that have not yet been fully elucidated. Chronic inflammation of the airways and lung parenchyma serves as a central driving factor, primarily mediated by infiltrating activated macrophages, neutrophils, CD8^+^ T lymphocytes, and other immune cells. These cells release large amounts of pro-inflammatory cytokines [e.g., tumor necrosis factor-α (TNF-α), interleukin-1β (IL-1β), interleukin-6 (IL-6), and interleukin-8 (IL-8)], chemokines [e.g., CXCL8、CXCL1/2, and proteases (e.g., neutrophil elastase (NE) and matrix metalloproteinases (MMPs)], which directly or indirectly cause lung tissue damage, leading to both localized (centrilobular) and widespread (panlobular) emphysema (2–4). Severe imbalance in the protease/antiprotease system and oxidative stress are key inflammatory processes contributing to COPD pathogenesis. On one hand, there is excessive production and activation of proteases such as NE and MMPs (e.g., MMP-9, MMP-12) (5–7); on the other hand, crucial antiproteases including α1-antitrypsin (AAT) and secretory leukocyte protease inhibitor (SLPI) are functionally deficient due to genetic defects in α1-AT, excessive consumption by proteases, or inactivation by oxidative stress, predisposing individuals to early-onset emphysema (8, 9). Epigenetic regulation acts as an important upstream mechanism, dynamically modulating the expression of key genes involved in inflammation, oxidative stress, protease production, and tissue remodeling through DNA methylation, histone modifications (e.g., acetylation, methylation), and non-coding RNAs (e.g., miRNAs), significantly influencing disease susceptibility and progression (10, 11). These mechanisms do not operate in isolation but are closely intertwined and mutually reinforcing: inflammation drives protease release and oxidative stress; oxidative stress exacerbates inflammation and impairs antiprotease function; and epigenetic alterations profoundly affect the gene expression profiles governing all these processes. However, the high complexity and individual heterogeneity of COPD pathogenesis imply that many critical questions remain unresolved. A deeper understanding of these interactive networks and undefined mechanisms is essential for developing more effective prevention and treatment strategies.

Experimental models are essential tools for elucidating the pathological mechanisms of COPD. Currently, the primary experimental models of COPD include cigarette smoke (CS) exposure, air pollution-induced models, and COPD exacerbation models (12). Among these, CS exposure, through the long-term inhalation of smoke particles and toxic substances, can stably induce chronic airway inflammation, alveolar destruction, and airflow limitation, making it the most widely utilized model that most closely mimics the COPD progression in the human smoking population in murine studies (13, 14). Air pollution-induced models primarily use particulate matter (such as PM2.5) or ozone exposure to trigger persistent airway inflammation and oxidative stress, reflecting the role of environmental factors in the onset and progression of COPD in rodent models (15–17). Additionally, viral or bacterial infection combined with CS exposure is often used to establish exacerbation models, which can replicate the amplified inflammation and immune imbalance seen in clinical patients with underlying pathological changes in experimental animal models (18), whereas clinical evidence from COPD patients has similarly demonstrated infection-associated inflammatory amplification (19). These models not only simulate pathological alterations under different etiologies but also provide important tools for revealing the dynamic changes in immune cells during disease initiation, progression, and exacerbation. The spatiotemporal evolution of immune cells is closely associated with inflammatory amplification, tissue damage, and treatment response. Therefore, a systematic elucidation of the spatiotemporal dynamics of immune cells in experimental COPD models is of great significance for unraveling the mechanisms of inflammatory amplification, tissue damage, and therapeutic responses, and for advancing clinical translational research.

## 2 Dynamic changes of immune cells in experimental COPD models

The progression of COPD is accompanied by complex immune cell infiltration and functional remodeling of immune responses. Different immune cells play distinct roles in the initiation of inflammation, tissue destruction, and disease exacerbation, and their dynamic changes are key to understanding disease mechanisms. Recent causal inference studies have established a causal relationship between immune cells and COPD (20). A subsequent comprehensive analysis using Mendelian randomization identified 41 immune cell phenotypes with causal links to the disease. Among these, six phenotypes exhibited evidence of reverse causality: CD14^+^CD16^+^ monocytes (absolute count), CD4^+^CD8^dim T cells expressed as a proportion of lymphocytes, CD4^+^CD8^dim T cells expressed as a proportion of total leukocytes, CD3^−^ lymphocytes (absolute count), effector memory CD8^bright T cells characterized by CD3 expression, and immature myeloid-derived suppressor cells characterized by CD45 expression. Furthermore, the study identified that the effects of eight specific immune phenotypes are mediated by eight metabolites, namely 1-palmitoyl-GPG (16:0), α-tocopherol, α-hydroxyisovalerate, 1-methylnicotinamide, cinnamoylglycine, taurochenodeoxycholate, N-oleoylserine, and X-19438 (21). These findings not only deepen our understanding of the role of immune cells in COPD but also provide new insights for screening high-risk populations, early disease prevention, and precise diagnosis.

### 2.1 Macrophages

Macrophages are among the first immune cells activated following CS exposure. Their numbers increase significantly in the lung tissues of both COPD patients and animal models, and they undergo polarization as the disease progresses (22, 23). Substantial evidence indicates that aberrant macrophage polarization is a key mechanism underlying sustained chronic inflammation, lung tissue destruction, and disease progression in COPD. During the acute exposure phase, cigarette smoke extract (CSE) drives alveolar macrophages toward an M1 phenotype through oxidative stress– and pattern recognition receptor–dependent mechanisms. Components of CSE activate toll-like receptors (particularly TLR4) and induce reactive oxygen species (ROS) production, leading to the activation of downstream NF-κB and MAPK signaling pathways that promote M1 polarization (24, 25). In this process,M1 macrophages markedly upregulate and secrete classical pro-inflammatory cytokines such as TNF-α and IL-1β (26), accompanied by high expression and release of MMP-12 (27). This protease is considered to play a critical role in elastin degradation and lung tissue damage, thereby promoting the onset and progression of COPD. Studies have indicated that in CS-induced COPD mouse models, MMP-12 levels positively correlate with the extent of alveolar structural destruction (27). Accordingly, macrophages in COPD exhibit pronounced functional heterogeneity, with distinct polarization states characterized by specific inducing signals, effector mediators, and pathological roles, as summarized in **Table 1**.

**Table 1:** Characteristics and roles of macrophage phenotypes in COPD.

Phenotype	Primary inducers	Key secreted factors	Primary functions	Role in COPD pathogenesis
M1 (Classically activated) (141) (142)	LPS, IFN-γ, GM-CSF	TNF-α, IL-6, IL-8, ROS, MMP-9, MMP-12	Pro-inflammatory; host defense	Drives chronic airway inflammation and emphysematous tissue destruction via excessive cytokine and protease release.
M2a (Wound-healing) (28, 141)	IL-4, IL-13	TGF-β, CCL18, fibronectin, procollagen	Tissue repair; extracellular matrix deposition	Promotes tissue remodeling and collagen deposition; may contribute to airway remodeling and fibrotic responses in COPD.
M2b (Regulatory) (28, 141)	Immune complexes, TLR agonists	IL-1β, IL-6, IL-10, TNF-α	Immune regulation; Th2 modulation	Modulates inflammatory responses; less well characterized in COPD, but may participate in immune regulation under specific inflammatory conditions.
M2c (Deactivating) (28, 141)	IL-10, glucocorticoids	IL-10, TGF-β, MMP-9	Anti-inflammatory; efferocytosis	Clears apoptotic cells and limits inflammation; impaired efferocytosis contributes to secondary necrosis and sustained inflammation in COPD.
M2d (Angiogenic) (28)	IL-6, adenosine	VEGF, TGF-β, IL-10	Angiogenesis; vascular remodeling	May contribute to vascular remodeling and altered microvascular structure in COPD lungs.

**Figure 1:**
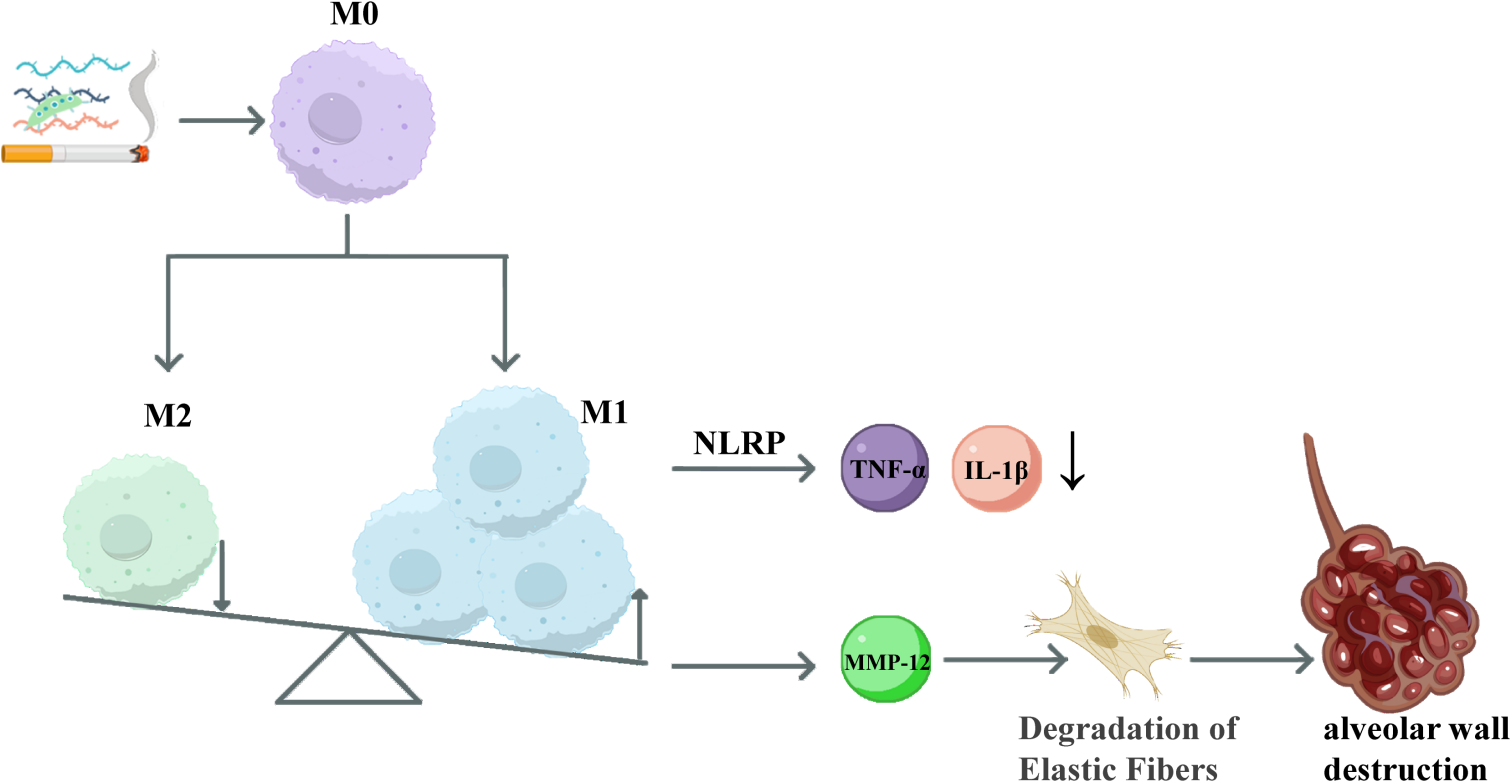
Dynamic schematic diagram of macrophages in COPD. Cigarette smoke shifts macrophages from an M0 state toward M1 polarization with a relative reduction in M2. M1 macrophages generate TNF-α and IL-1β via the NLRP inflammasome and secrete MMP-12 to degrade elastin, leading to alveolar wall destruction.

However, in the chronic inflammatory environment, the M1/M2 balance of macrophages is persistently disrupted, characterized by a predominance of the M1 phenotype and impaired function of the M2 phenotype. It is noteworthy that M2 subtypes (M2a, M2b, M2c, M2d) may not only promote tissue repair but also drive airway remodeling and fibrosis at different stages of the disease, thereby exhibiting a “double-edged sword” effect in disease progression (28). The establishment and maintenance of macrophage polarization are regulated by multiple signaling pathways and metabolic states. M1 polarization is primarily driven by pro-inflammatory transcription factors such as NF-κB, STAT1, and IRF5 (29–32), while M2 polarization relies on the activation of STAT6 and IRF4 (33), and is subject to negative feedback control of the JAK/STAT and PI3K pathways by SOCS proteins (particularly SOCS1 and SOCS3). For instance, loss of SOCS3 can enhance the M2 phenotype (34). Concurrently, studies indicate that the JNK pathway plays a dual role in macrophage polarization (35–37). Meanwhile, CS-induced oxidative stress can activate the NLRP3 inflammasome in M1 macrophages, further enhancing the release of pro-inflammatory cytokines such as IL-1β and exacerbating the inflammatory response (38, 39). A dynamic schematic diagram of macrophages is shown in **Figure 1**.

At the metabolic level, M1 macrophages rely on glycolysis for energy production, whereas M2 macrophages depend on fatty acid oxidation and mitochondrial oxidative phosphorylation (40, 41). In the hypoxic and oxidative stress microenvironment characteristic of COPD, metabolic dysregulation in macrophages favors a shift toward the M1 phenotype, thereby sustaining the chronic inflammatory state (42, 43). Beyond intrinsic signaling, macrophage phenotype specification is also influenced by intercellular interactions. For instance, epithelial cell-derived exosomal miR-125a-5p transported via epithelial cell-derived exosomes to macrophages can promote M1 polarization by targeting interleukin-1 receptor antagonist (IL1RN), while basophil-secreted IL-4 induces MMP-12 production in macrophages, accelerating alveolar structural destruction (44, 45). As the disease progresses chronically, macrophage function gradually exhibits a “dual imbalance.” On one hand, their ability to clear apoptotic cells and pathogens via efferocytosis is impaired, manifesting as suppressed LC3-associated phagocytosis (46). On the other hand, metabolic defects such as mitochondrial dysfunction and enhanced oxidative stress further compromise their immune effector functions (47, 48). Concurrently, in addition to MMP-12, proteases such as MMP-9 and elastase contribute to alveolar destruction. Normally, α1-antitrypsin acts as a critical inhibitor of neutrophil elastase to preserve lung tissue integrity; thus, its deficiency exacerbates the protease/antiprotease imbalance, driving the development and progression of emphysema (49, 50).

### 2.2 Neutrophils

Neutrophils are the most prominently infiltrating granulocytes in COPD, and their numbers positively correlate with the intensity of airway inflammation and the rate of lung function decline, surging dramatically during acute exacerbations (51, 52).

#### 2.2.1 Mechanisms of neutrophil recruitment in early inflammatory responses in COPD

**Figure 2:**
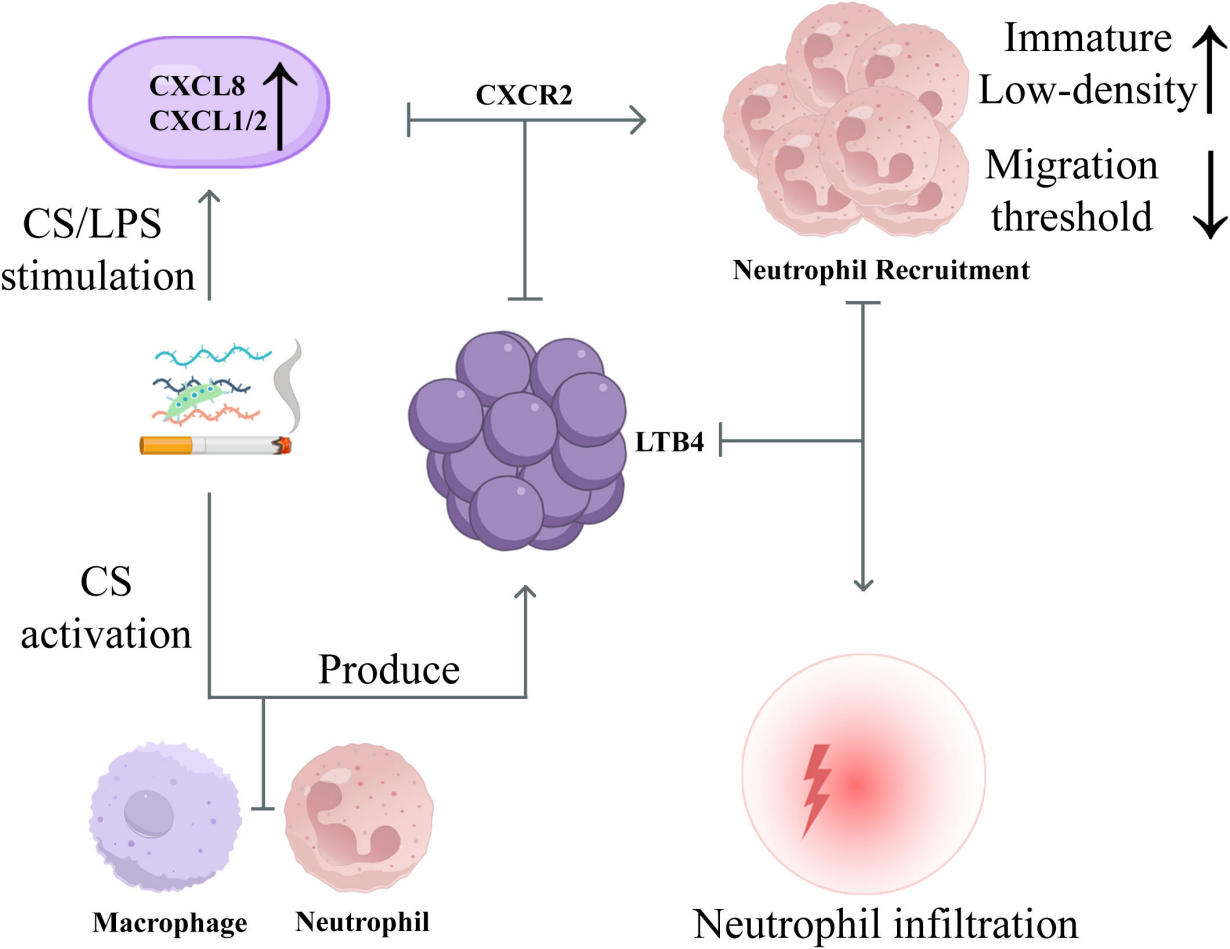
Schematic diagram of neutrophils in COPD during the inflammatory initiation phase. During inflammation initiation, CS/LPS increases CXCL8 and CXCL1/2 and recruits neutrophils through CXCR2. Signals such as LTB4 lower the migratory threshold and promote infiltration of immature low-density neutrophils.

During the initial phase of inflammation, CS or pathogen-associated stimuli can induce airway epithelial cells to release chemokines such as CXCL8 and CXCL1/2, while simultaneously activating innate immune cells including macrophages and neutrophils to produce inflammatory mediators like LTB_4_. These factors collectively drive the recruitment and sustained migration of neutrophils via the CXCR2 axis (53–55). Concurrently, the proportion of immature or low-density neutrophils increases in the bone marrow and peripheral blood, resulting in a corresponding reduction in their migration threshold (56). Studies have shown that stimulation with CS or lipopolysaccharide (LPS) can promote airway epithelial cells to secrete CXCL8, thereby recruiting large numbers of neutrophils to infiltrate the lung tissue (54). In mouse models exposed to CS, the number of neutrophils in bronchoalveolar lavage fluid (BALF) is significantly elevated, and this high level of infiltration can persist with prolonged exposure (57), indicating that neutrophil recruitment is a critical feature of the early inflammatory response in COPD (58). The schematic diagram of neutrophils during the inflammatory initiation phase is shown in **Figure 2**.

#### 2.2.2 Pathogenic roles in tissue injury

**Figure 3:**
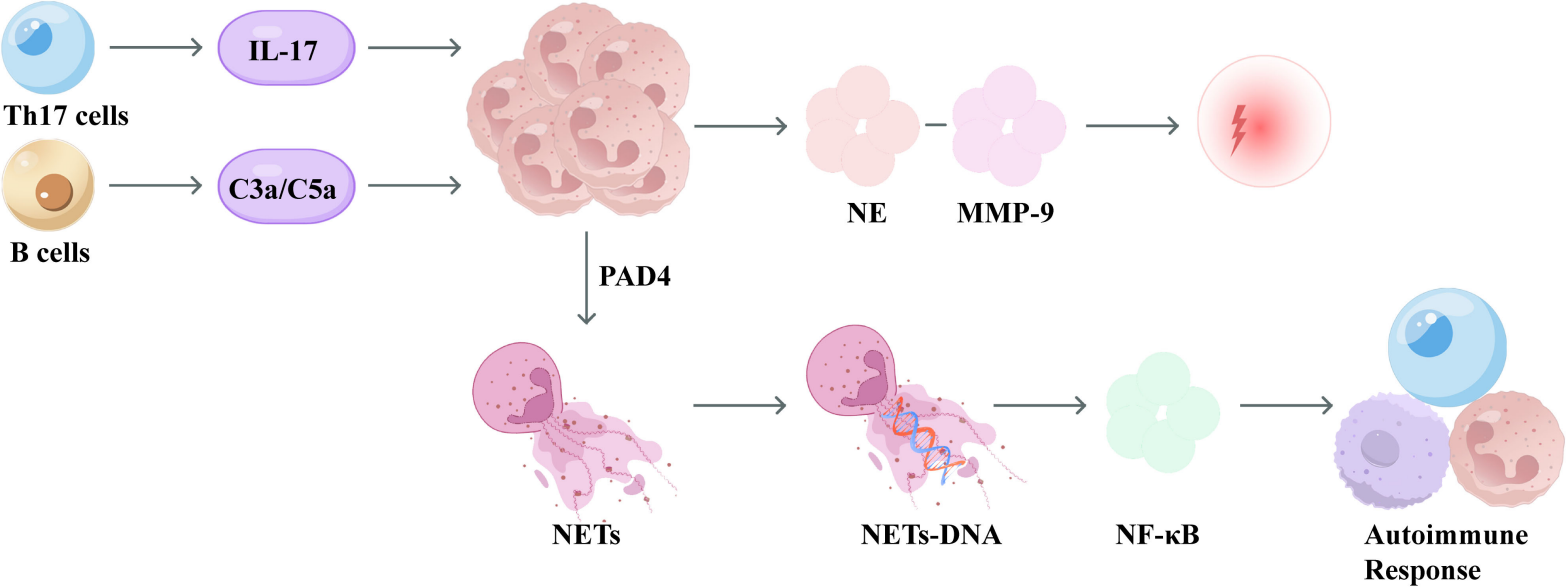
Schematic diagram of neutrophils in COPD during the tissue damage phase. In the tissue-damage phase, Th17-derived IL-17 and complement C3a/C5a enhance neutrophil activation. Neutrophil elastase (NE) and MMP-9 drive tissue injury, while PAD4 promotes NET formation; NET-derived DNA activates NF-κB and contributes to autoimmune-like responses.

During the tissue damage phase, neutrophils exacerbate lung tissue destruction primarily through two mechanisms. The first is direct damage: neutrophils release proteases such as elastase and MMP-9, which directly degrade the alveolar matrix (59). The second mechanism involves indirect tissue damage: DNA derived from neutrophil extracellular traps (NETs), generated during PAD4-dependent NET formation, can activate NF-κB–dependent autoimmune responses via the cGAS–TLR9 pathway, thereby perpetuating chronic airway inflammation (60). Infiltrating Th17 cells indirectly regulate the recruitment of neutrophils through the secretion of IL-17 (61), while Infiltrating B cells promote neutrophil recruitment in lung tissue via the formation of immune complexes, which subsequently activate complement components C3a and C5a (62). At the metabolic level, neutrophils exhibit a shift toward heightened glycolysis and demonstrate a HIF-1α-driven “long-lived” phenotype. Concurrently, oxidative stress leads to reduced HDAC2 levels, resulting in glucocorticoid insensitivity (63). In animal models of COPD, NET-derived DNA levels positively correlate with the extent of alveolar enlargement, and degradation of NETs by DNase significantly alleviates airway inflammation (60). Furthermore, clinical studies have observed that elevated serum levels of HIF-1α and IL-19 in COPD patients are closely associated with disease progression (63). The schematic diagram of neutrophils during the tissue damage phase is shown in **Figure 3**.

#### 2.2.3 Impaired resolution of inflammation

**Figure 4:**
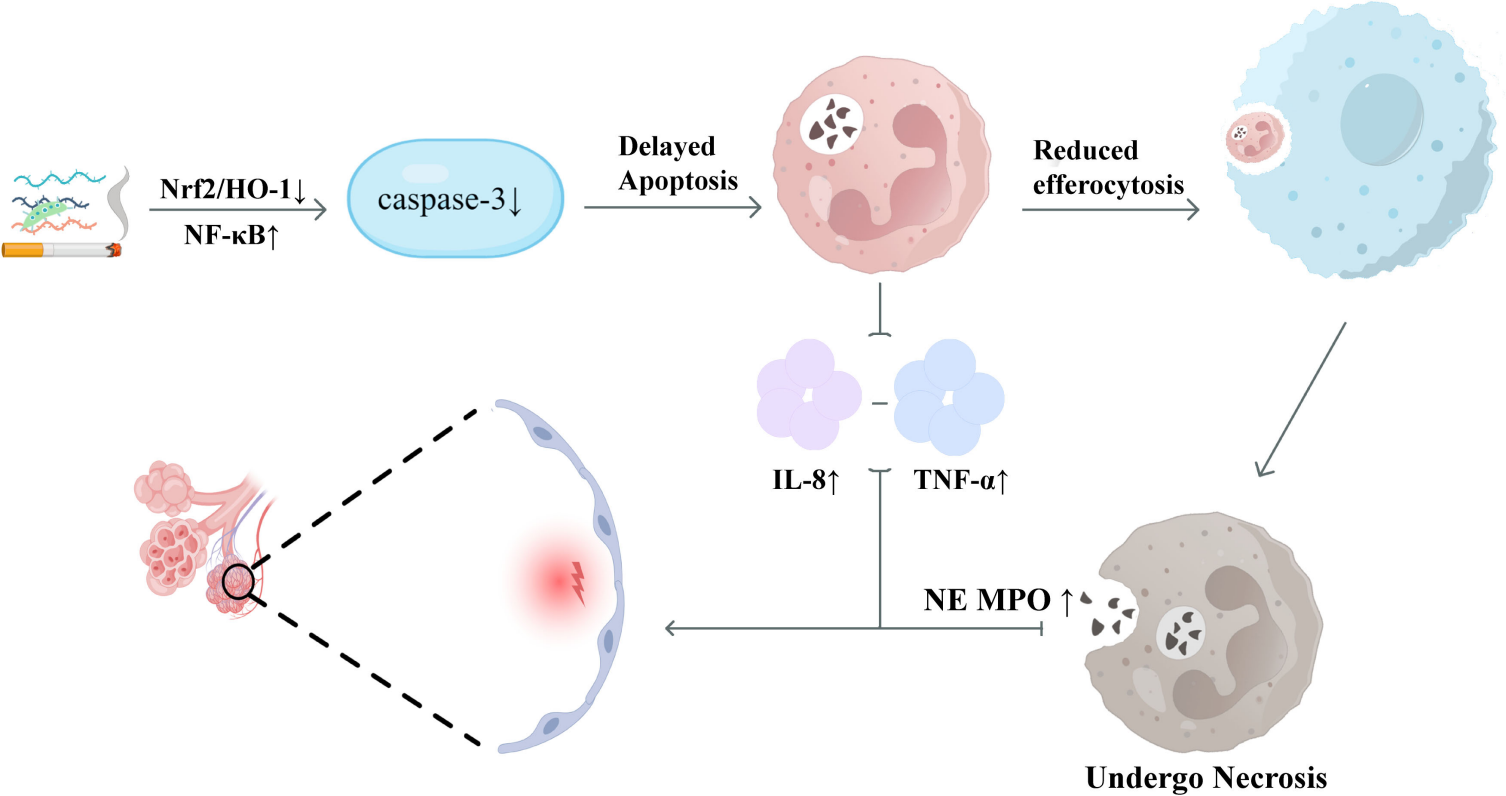
Schematic diagram of neutrophils in COPD during the inflammatory resolution phase. During inflammation resolution, decreased neutrophil caspase-3 delays apoptosis and impaired macrophage clearance leads to neutrophil retention. Persisting neutrophils are prone to necrosis and release NE/MPO, while elevated IL-8 and TNF-α sustain chronic inflammation.

Programmed apoptosis of neutrophils is a critical initiating point for inflammation resolution: following apoptosis, neutrophils can be cleared by macrophages through efferocytosis, thereby preventing the release of tissue-damaging molecules such as neutrophil elastase (NE) (64) and myeloperoxidase (MPO) (65), and averting further spread of inflammation. However, in COPD, this mechanism is significantly impaired. Studies have shown that the spontaneous apoptosis rate of neutrophils in the peripheral blood and BALF of COPD patients is reduced, accompanied by an upregulation of anti-apoptotic factors. Pathogenic factors such as cigarette smoking can activate the NF-κB pathway and inhibit Nrf2/HO-1 signaling, thereby reducing the activity of the apoptosis-related protein Caspase-3 (66), leading to delayed neutrophil apoptosis and prolonged survival. These long-lived neutrophils not only continuously secrete pro-inflammatory cytokines such as IL-8 and TNF-α (67) but also, due to delayed apoptosis, impair the efferocytic efficiency of macrophages. This results in secondary necrosis of uncleared cells, further exacerbating the inflammatory response and tissue damage (67, 68). The schematic diagram of neutrophils during the inflammatory resolution phase is shown in **Figure 4**.

Furthermore, neutrophils can synthesize anti-inflammatory lipid mediators via the lipoxygenase (LOX) pathway, including lipoxin A_4_ (LXA_4_) and resolvins. Among them, LXA_4_ inhibits neutrophil chemotaxis and the release of pro-inflammatory cytokines from macrophages by binding to the ALX/FPR2 receptor (69). Resolvins, on the other hand, directly inhibit the activity of NE and MPO within neutrophils, thereby alleviating oxidative stress damage (70). Therefore, the declined capacity of neutrophils to synthesize these anti-inflammatory mediators is considered one of the key mechanisms contributing to ineffective inflammation resolution and continuous disease progression in COPD.

#### 2.2.4 Dysregulation of neutrophil function in the tissue repair phase

During the tissue repair phase, pulmonary tissue repair relies on the finely coordinated processes of “epithelial regeneration—mesenchymal repair—structural remodeling.” Under normal conditions, neutrophils play a positive role in early injury repair: they secrete platelet-derived growth factor (PDGF) to recruit fibroblasts and promote collagen synthesis; release vascular endothelial growth factor (VEGF) to facilitate capillary angiogenesis; and produce epidermal growth factor (EGF) to stimulate the proliferation of airway epithelial cell (71). Simultaneously, neutrophils release MMP-8 and MMP-9 to moderately degrade the extracellular matrix (ECM), thereby creating space for the migration of repair cells (72).

**Figure 5:**
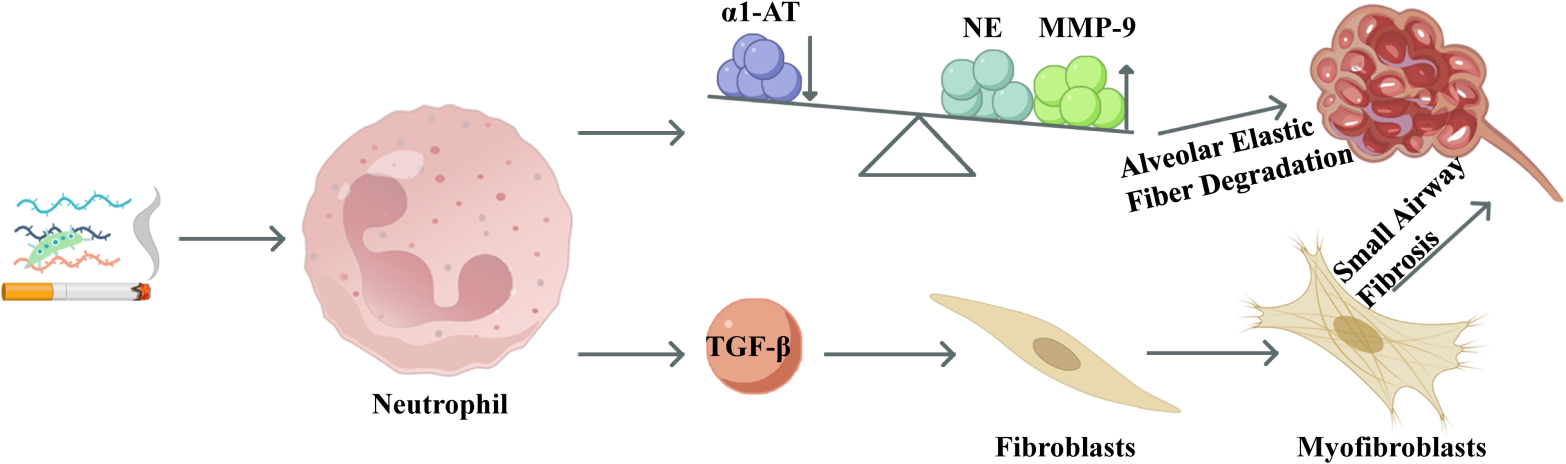
Schematic diagram of neutrophils in COPD during the tissue repair phase. In the tissue-repair phase, α1-antitrypsin deficiency allows NE and MMP-9 to dominate, accelerating elastin degradation. Neutrophil-derived TGF-β activates fibroblasts and promotes myofibroblast differentiation, driving small-airway fibrosis and remodeling.

However, in COPD, the repair process becomes aberrant, and neutrophil dysfunction emerges as a central driving factor. On one hand, excessive release of NE and MMP-9 by neutrophils, coupled with significant smoking-induced suppression of antiprotease activity (e.g., α1-antitrypsin), leads to a disruption of the protease/antiprotease balance (73). This imbalance results in excessive degradation of alveolar elastic fibers, contributing to the development of emphysema (74). On the other hand, overexpression of transforming growth factor-beta (TGF-β) by neutrophils hyperactivates fibroblasts, promoting their differentiation into myofibroblasts and driving the irreversible formation of small airway fibrosis (75). The schematic diagram of neutrophils during the tissue repair phase is shown in **Figure 5**.

Multiple strategies have been explored to target neutrophil dysfunction. Commonly used agents, such as long-acting anticholinergics, PDE4 inhibitors, and macrolide antibiotics, can inhibit neutrophil migration, degranulation, or pro-inflammatory cytokine release to varying degrees, thereby mitigating inflammatory damage (76–78). Additionally, novel therapeutic approaches including CXCR2 antagonists, NE inhibitors, and pro-resolving mediators (e.g., LXA_4_, Resolvin E1) have shown promising potential (69, 79). Therefore, targeting neutrophils is considered a crucial direction for COPD intervention, and the clinical application prospects of related therapies warrant further investigation.

### 2.3 T cells

In experimental models of COPD, T cells are recognized as pivotal adaptive immune effector cells that contribute to the sustained chronic inflammation and tissue remodeling. In contrast to the acute inflammation predominantly driven by neutrophils, T cells confer the disease with its persistent and refractory nature through mechanisms including immunological memory, cytotoxicity, and long-term modulation of cytokine networks. Importantly, chronic antigen exposure in COPD also drives T-cell metabolic dysfunction, immunosenescence, and dysregulated immune checkpoint signaling, which together contribute to persistent inflammation and impaired immune resolution (80).

#### 2.3.1 CD8^+^ T cells

In CS mouse models, CD8^+^ T cells extensively infiltrate the lung parenchyma and airway mucosa, and their levels show a negative correlation with declining lung function (81, 82). Activated CD8^+^ T cells release perforin and granzyme B, which directly mediate the apoptosis of alveolar and airway epithelial cells, thereby promoting alveolar destruction and small airway narrowing (83, 84). Furthermore, CD8^+^ T cells secrete IFN-γ and TNF-α, which further amplify inflammatory responses by enhancing macrophage and neutrophil activation, thereby establishing a bidirectional loop of cytotoxicity-amplified inflammation (82). With prolonged stimulation, COPD-associated CD8^+^ T cells may acquire features of senescence and partial exhaustion, characterized by sustained cytotoxic activity but reduced proliferative capacity, further exacerbating tissue damage while limiting effective immune regulation (85).

#### 2.3.2 CD4^+^ T cells

In studies of COPD models, the subset differentiation of CD4^+^ T cells exhibits a significant bias, which is closely associated with the chronic inflammatory progression of the disease. Specifically, infiltrating Th1 cells enhance macrophage activity through the secretion of interferon-γ (IFN-γ), thereby sustaining the persistent activation of inflammatory cascades (86). Similarly, infiltrating Th17 cells secrete interleukin-17A/F (IL-17A/F), which upregulates the expression of the chemokine CXCL8 and promotes neutrophil recruitment, serving as a critical driver for the maintenance of chronic inflammation and the occurrence of acute exacerbations in COPD (87–89). In contrast, pulmonary regulatory T cells (Tregs), which possess immunosuppressive functions, are not only reduced in number but also exhibit significant functional impairment in COPD. This is characterized by insufficient expression of forkhead box protein 3 (Foxp3) and reduced secretion of interleukin-10 (IL-10), ultimately leading to diminished immunosuppression and impaired resolution of inflammation (90). Current evidence indicates that a disrupted immune equilibrium — characterized by an elevated Th1 response, a dysregulated Th17 response (involving both chronic activation and acute functional impairment), and defective Treg-mediated immunosuppression — constitutes a pivotal mechanism driving persistent inflammation and disease progression in COPD (91–93).

#### 2.3.3 Tissue-resident memory T cells

Smoke exposure can induce the generation of tissue-resident memory T cells (TRM) in the lungs, which rapidly produce cytokines upon re-stimulation, thereby sustaining a pro-inflammatory tone (94). The lungs of COPD patients exhibit an enrichment of CD8^+^ TRM cells, which are associated with IFN-γ signaling, impairment of epithelial stem-progenitor cell pools, and small airway remodeling (22). Furthermore, TRM cells can promptly release cytokines in response to recurrent stimuli such as pathogens or pollutants, maintaining a state of low-grade inflammatory “noise” (95). This “resident-reactivation” model provides an immunological explanation for the chronicity of COPD. Repeated reactivation of lung TRM cells in COPD is also linked to metabolic stress and progressive functional aging, suggesting a potential role for immunosenescence and immune checkpoint regulation in sustaining chronic inflammation.

#### 2.3.4 Therapeutic interventions and T cell-related targets

Targeting the IL-17/IL-23 axis represents one of the most consistent T-cell pathway strategies; inhibition of IL-17A or blockade of upstream IL-23 has been shown to reduce inflammation and structural damage in multiple mouse models (96, 97). In parallel, strategies focused on Treg restoration offer a complementary approach. For instance, IL-2 immunocomplexes (IL-2C) can selectively induce Treg expansion, and low-dose IL-2 complexes in smoke and LPS models have demonstrated the potential to alleviate COPD progression by restoring immune balance and reducing inflammation (98). Beyond specific cell subsets, modulating Tissue-Resident Memory (TRM) T cells—specifically their maintenance, migration, and IFN-γ signaling axis—may reduce the ‘chronic inflammatory noise’ caused by repeated activation (95). At the cellular metabolic level, given that mTOR/glycolysis pathways critically determine T-cell differentiation and effector functions (99), metabolic reprogramming tailored to the COPD-specific microenvironment (e.g., hypoxia, oxidative stress) (100), warrants clinical exploration, potentially in combination with IL-17 axis modulation (101).Finally, regarding immune checkpoints, PD-1/PD-L1 signaling in COPD exhibits a complex pattern of coexisting activation and exhaustion. This suggests a need for precise modulation rather than simple inhibition or activation (102), and further mechanistic studies are required to carefully balance infection risks with anti-tumor immunity.

**Figure 6:**
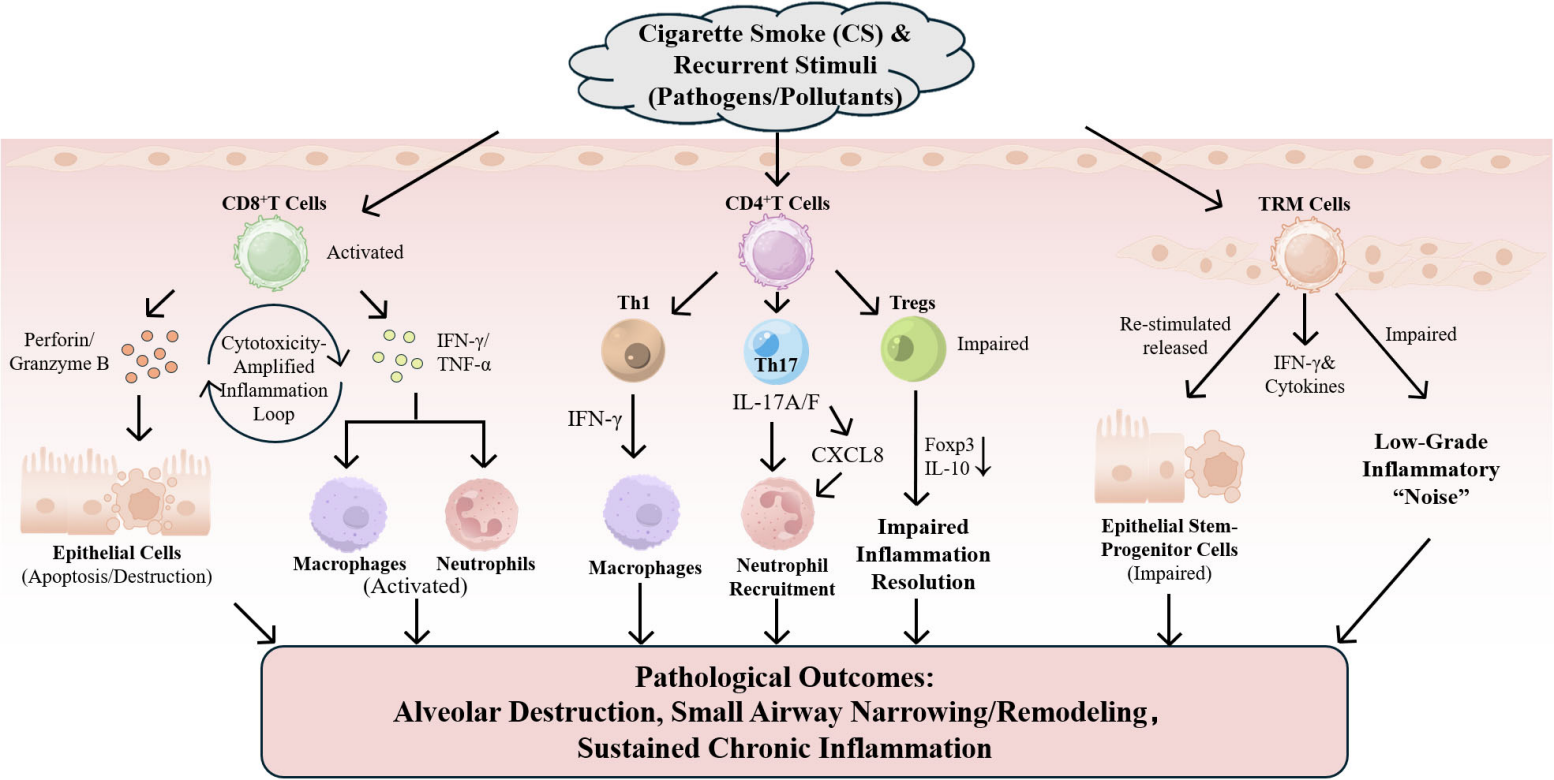
T cell-driven mechanisms in COPD pathology. Cigarette smoke and recurrent stimulation drive T cell–related mechanisms: CD8+ T cells mediate epithelial injury and amplify inflammation; CD4+ T cells skew toward Th1/Th17 responses with reduced Treg function; TRM cells continuously release IFN-γ and other cytokines upon re-stimulation and impair epithelial stem/progenitor cell function, ultimately leading to alveolar destruction, small-airway narrowing/remodeling, and persistent chronic inflammation.

In summary, T cells shape the chronic immune ecology and tissue remodeling in COPD through multiple mechanisms, including cytotoxicity, cytokine networks, tissue residency, and metabolic-senescence axes. Combinatorial immunomodulatory strategies targeting the IL-17/IL-23 axis, Treg restoration, TRM maintenance, and metabolic-checkpoint co-regulation represent key future directions for moving beyond “inflammatory relief” toward “modifying disease progression”. An overview of the key T cell–mediated mechanisms involved in COPD pathology is presented in **Figure 6**.

### 2.4 Other immune cells

#### 2.4.1 Fibrocytes

In the CS+LPS-induced lung injury model, fibrocytes are recruited to lung tissue through specific mechanisms. Originating from the bone marrow, these cells enter the peripheral circulation and traffic to the lungs guided by chemotactic signals. This recruitment is primarily mediated by the CXCL8–CXCR1/2 axis—driven particularly by CXCL8 secreted from activated CD8^+^ T cells—and CXCR4-dependent pathways, which are notably active during acute exacerbations. Upon arrival, activated fibrocytes secrete various chemokines (e.g., CCL2, CCL3, CXCL1) and mediators (e.g., TGF-β, MMPs). Through these secreted factors and direct cell-to-cell contact (via CD54/CD86), they further activate CD8^+^ T cells. In turn, activated CD8^+^ T cells release cytokines such as TNF-α and IFN-γ, which promote fibrocyte proliferation, survival, and immunophenotypic conversion. This reciprocal interaction establishes a sustained ‘inflammation–fibrosis’ positive feedback loop, thereby exacerbating airway remodeling and disease progression (103). Studies have shown that elevated circulating fibrocyte counts during acute exacerbations of COPD are associated with increased mortality risk, suggesting that fibrocytes may contribute detrimentally to disease progression. Recruited fibrocytes may participate in pulmonary inflammation due to their immunogenic properties (104).

#### 2.4.2 Natural killer cells

In a cigarette smoke-induced murine model, chronic CS exposure can predispose natural killer (NK) cells to a “pre-activated” state, leading to an increased release of IFN-γ upon viral infection and further exacerbating pulmonary inflammation (105). In clinical studies involving COPD patients, the expansion of the adaptive NKG2C^+^ NK cell subset in models shows a positive correlation with the frequency of acute exacerbations, suggesting its potential as a biomarker for predicting exacerbation risk (106). Furthermore, regarding cytokine regulation in smokers, CS exposure exerts differential effects on interleukin-16 (IL-16) concentrations: long-term smoking significantly increases extracellular IL-16 released by locally recruited CD4^+^ cells in the airways, yet does not markedly alter systemic IL-16 levels in peripheral blood. Notably, during this process, intracellular IL-16 concentrations in blood NK cells exhibit a significant decrease. This distinct pattern of “local elevation alongside intracellular reduction” may reflect the localized regulatory impact of CS on IL-16 metabolism (107). Functionally, as a potent chemoattractant for CD4^+^ immune cells, the accumulation of IL-16 in the airways facilitates the recruitment of inflammatory cells (such as CD4^+^ T cells and macrophages), thereby perpetuating the chronic inflammatory response characteristic of COPD (108).

#### 2.4.3 Eosinophils and basophils

**Figure 7:**
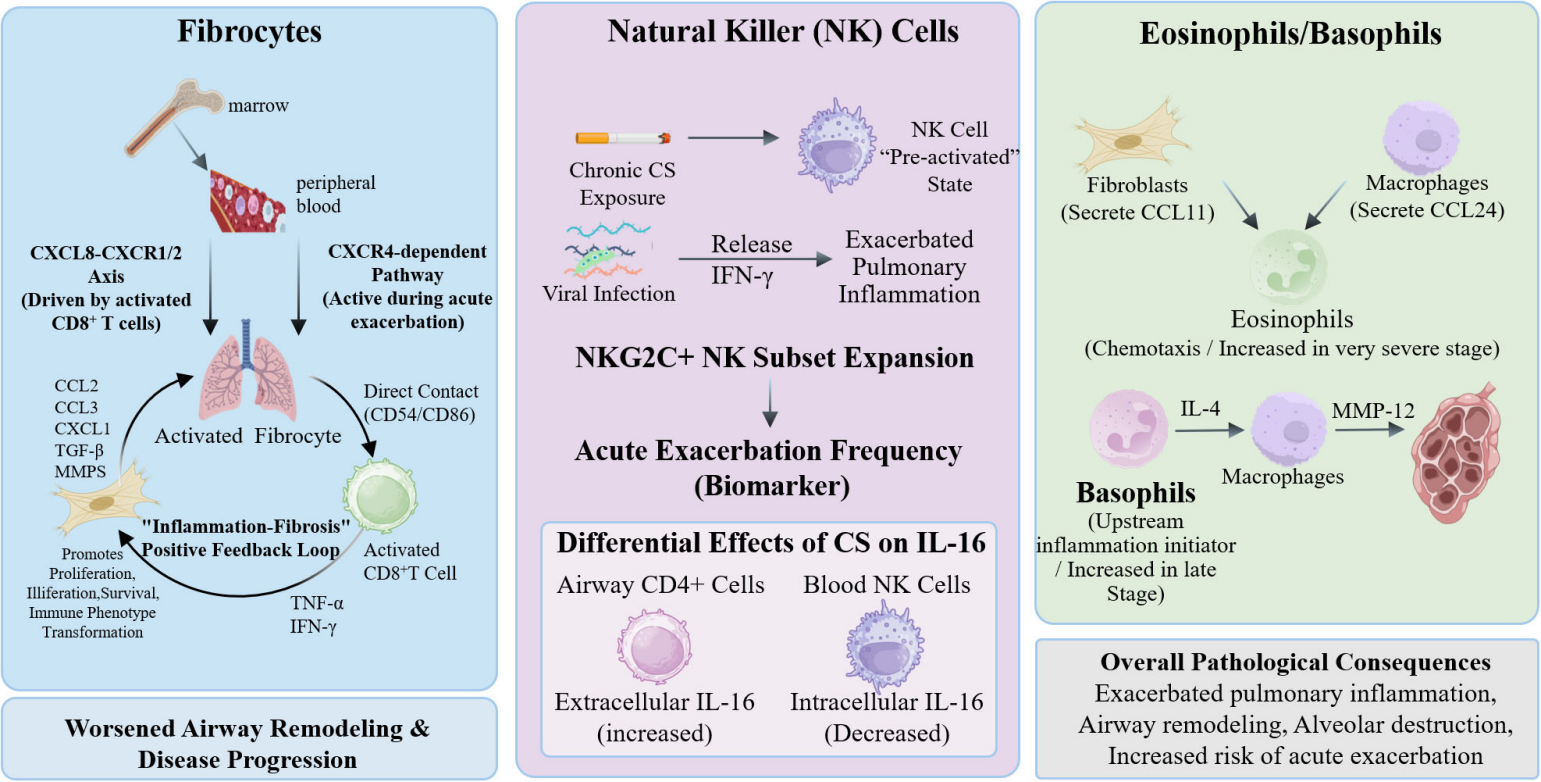
Mechanisms of other immune cells in COPD models fibroblasts are recruited to the lung via CXCL8–CXCR1/2 and CXCR4 signaling and interact with CD8+ T cells, forming a positive feedback loop between inflammation.

Eosinophils and basophils are distributed across various anatomical regions of the lungs in COPD patients and are significantly increased in very severe stages of the disease (109). Studies have shown that spatially restricted eosinophil chemotactic signals, such as CCL11 secreted by fibroblasts and CCL24 produced by macrophages, can be observed in both murine models and human lung tissues. Furthermore, increased tissue basophil infiltration has been identified as a novel feature of advanced COPD (109). In an elastase-induced emphysema model, basophils promote alveolar destruction by secreting IL-4, which induces macrophage production of MMP-12. Notably, basophil-deficient mice exhibit attenuated emphysema compared to wild-type mice, confirming the role of basophils as an “upstream initiator” of inflammation (44). **Figure 7** summarizes the major pathogenic mechanisms driven by other immune cell types in experimental models of COPD.

## 3 Dynamic changes in immune cells and treatment response in COPD headings

The Systemic Immune-Inflammation Index (SII) is a quantitative measure of systemic immune-inflammatory responses in the human body. A population-based study by Ye et al. (110) found that, after adjusting for all other factors, a higher SII level was independently associated with an increased likelihood of COPD (OR = 1.449; 95% CI: 1.252–1.676, P < 0.0001). Furthermore, COPD patients with elevated SII levels faced a higher risk of all-cause mortality. This indicates a close relationship between systemic inflammation and both the onset and progression of COPD. Inflammation manifests in diverse forms, reflected in heterogeneity among different airway inflammatory patterns or airway microbial ecology. Among these, the most common inflammatory phenotype is neutrophil-associated COPD with inflammasome, Th1, and Th17 immune activation, while a minority of patients exhibit eosinophil-associated T2-mediated immunity (111).

### 3.1 Treatment of COPD based on the eosinophil-associated T2-mediated inflammatory phenotype

In anti-inflammatory therapy, early research has identified specific targets including the glucocorticoid receptor cAMP and specific cyclic nucleotide phosphodiesterases (PDE4). Corticosteroids are most effective in patients with evidence of eosinophilic inflammation and have been used for over 40 years in the treatment of COPD, demonstrating modest overall benefits in improving lung function, health status, 6-minute walking distance, and reducing the frequency of acute exacerbations (112). The Global Initiative for COPD recommends blood eosinophil counts as a biomarker to guide the use of inhaled corticosteroids in COPD patients with frequent exacerbations (112). Furthermore, recent studies have demonstrated that corticosteroids alter the phenotype and function of macrophages in COPD patients, while simultaneously inhibiting cytokine release from macrophages (113). Nevertheless, inhaled corticosteroids are associated with numerous non-inflammatory side effects, such as hyperglycemia, osteoporosis, cataracts, and impacts on childhood growth (114). Additionally, roflumilast, a selective PDE4 inhibitor, reduces systemic and pulmonary inflammation in patients with severe COPD and improves disease symptoms. It is indicated for add-on therapy in COPD patients inadequately controlled by dual bronchodilators (LAMA + LABA). Results from a 16-week randomized controlled trial showed that, compared to placebo, roflumilast was associated with a significant reduction in eosinophils in bronchial biopsy specimens at week 16 (RR = 0.53; 95% CI: 0.34–0.82; p = 0.0046). Moreover, roflumilast also led to significant reductions in both absolute (p = 0.0042) and differential (p = 0.0086) eosinophil counts in induced sputum. The benefits of roflumilast may be mediated by the reduction in eosinophilic inflammation (115). Typical side effects of oral PDE4 inhibitors include nausea, diarrhea, and mood and behavioral changes, which significantly limit their use. Based on recent studies in COPD models, numerous novel therapeutic entities, particularly monoclonal antibodies targeting various key regulators of the immune system, have been routinely introduced into clinical practice. These agents primarily include those that selectively block specific targets involved in eosinophil, neutrophil, lymphocyte, and type 2 inflammatory pathways.

#### 3.1.1 Anti-IL-5 therapy

IL-5 is a major cytokine secreted by lymphocytes, eosinophils, and other cells. Several drugs targeting this pathway have been approved for clinical use, including anti-IL-5 monoclonal antibodies (mepolizumab and reslizumab) and an IL-5 receptor antibody (benralizumab). These agents have revolutionized the clinical management of IL-5-mediated severe asthma (116). However, in contrast to asthma, their efficacy in phase 2 and 3 trials for COPD has been disappointing. Studies of anti-IL-5 biologics in COPD have yielded mixed results regarding the reduction of exacerbation frequency, with no evidence demonstrating improvement in lung function, symptom relief, or enhanced quality of life—despite effectively depleting peripheral blood eosinophils (117).

In the first phase 2 trial of an anti-IL-5 receptor monoclonal antibody (benralizumab) for COPD, although a reduction in eosinophil counts and an improvement in forced expiratory volume in 1 second (FEV_1_) were observed in treated patients, benralizumab did not reduce the rate of acute exacerbations of COPD compared with placebo, and no differences in health status were found between the groups (118). Furthermore, two phase 3 clinical trials of benralizumab (GALATHEA and TERRANOVA) showed that none of the benralizumab doses significantly reduced the annualized rate of COPD exacerbations compared to placebo in patients with moderate to very severe COPD and blood eosinophil counts of 220 cells per cubic millimeter or higher.

Mepolizumab, an anti-IL-5 monoclonal antibody, was approved by the U.S. Food and Drug Administration (FDA) in 2015 as an add-on therapy for severe eosinophilic asthma (119). However, the results of two phase 3 trials (METREX and METREO) evaluating mepolizumab in COPD patients were very similar to those of benralizumab and failed to demonstrate satisfactory outcomes (117, 120).Another anti-IL-5 antibody, reslizumab, functions similarly to mepolizumab by preventing IL-5 from binding to the eosinophil surface. To our knowledge, reslizumab has not been formally evaluated in COPD patients. In a study involving patients with eosinophilic asthma, reslizumab did not significantly reduce exacerbations compared to placebo (p = 0.0833), but it did lead to a significant improvement in FEV_1_, with a mean increase of 0.24 L (p = 0.0023) (121).

#### 3.1.2 Anti-IL-13/IL-4 therapy

IL-4 and IL-13, both Th2 cytokines, are responsible for numerous functions in the development of COPD (122). IL-13 is produced by T cells, mast cells, basophils, and dendritic cells. It is involved in the regulation of inflammatory and immune responses, as well as in mucus hypersecretion (123). Dupilumab is a fully human monoclonal antibody that blocks the shared receptor component of IL-4 and IL-13. In a phase 3, multicenter, international, double-blind, randomized, placebo-controlled trial (the BOREAS trial) evaluating the efficacy and safety of dupilumab in patients with COPD, those receiving dupilumab showed significant improvement compared to those on placebo (RR = 0.70; 95% CI, 0.58–0.86; P < 0.001), along with better lung function, improved quality of life, and milder respiratory symptoms. This landmark study demonstrated satisfactory outcomes for type 2 inflammation-targeted therapy in COPD (124). A major strength of the BOREAS trial was its design as a highly powered international study with a strict exclusion criterion of a current or previous asthma diagnosis. Another phase 3, double-blind, randomized trial of dupilumab in COPD patients, published the following year, similarly showed that the dupilumab group had fewer COPD exacerbations (RR = 0.66; 95% CI, 0.54 to 0.82; P < 0.001) and better lung function compared to the placebo group (125). The incidence of adverse events was similar between the two groups. These studies indicate that dupilumab, as an add-on to standard triple therapy, exhibits substantial therapeutic potential and is, to date, the first targeted agent shown to unequivocally reduce COPD exacerbation rates and improve lung function. Tralokinumab is another monoclonal antibody targeting IL-13, which has been approved for the treatment of moderate-to-severe atopic dermatitis in adult patients who are candidates for systemic therapy (126). However, clinical evaluation of tralokinumab for COPD has been discontinued. Furthermore, no studies or clinical trial data are available regarding tralokinumab in COPD patients, which also limits its clinical application in this population.

#### 3.1.3 Anti-TSLP and Anti-IL-33 therapies

Tezepelumab is a human monoclonal antibody targeting thymic stromal lymphopoietin (TSLP). Mechanistically, TSLP is an epithelial cell-derived alarmin released in response to injury (e.g., cigarette smoke) that activates dendritic cells to drive Type 2 inflammation. Despite this theoretical rationale, a phase 2 trial investigating tezepelumab in adults with moderate-to-very severe COPD did not yield satisfactory results (127). Compared with placebo, the tezepelumab group showed no significant reduction in the annualized rate of moderate or severe COPD exacerbations (RR = 0.83; 90% CI, 0.64–1.06; p = 0.10). Further studies are needed to evaluate the efficacy of tezepelumab in patients with moderate-to-very severe COPD. Regarding other alarmins, itepekimab, which targets and blocks interleukin-33 (IL-33), has demonstrated anti-inflammatory activity in patients with asthma (128). Functionally, IL-33 is a nuclear cytokine of the IL-1 family released by damaged epithelial cells. It binds to the ST2 receptor on immune cells (such as ILC2s), triggering type 2 inflammatory pathways and mucus hypersecretion (129). In a phase 2a randomized controlled trial assessing the safety and efficacy of itepekimab in patients with moderate-to-severe COPD, itepekimab reduced exacerbation rates and improved lung function in former smokers with COPD (128). These encouraging results have led to the initiation of phase 3 studies to confirm the efficacy and safety of itepekimab in former smokers with COPD.

In summary, these findings suggest that eosinophils present in the airways may not play a major pathobiological role in COPD patients. More robust evidence of eosinophilic inflammation is required for the recruitment of future clinical trials.

### 3.2 Therapy for COPD with neutrophil-associated inflammatory phenotype

#### 3.2.1 Anti-TNF therapy

Several hypotheses have been proposed regarding the role of TNF-α in the pathogenesis of COPD. One major perspective suggests that TNF-α can activate inflammatory cells and plays a critical role in defending against various infectious pathogens (130). Infliximab, an anti-TNF-α antibody, was evaluated in a multicenter randomized controlled trial (RCT) involving patients with moderate to severe COPD, which demonstrated no additional clinical benefit (131). Another small-scale study assessed the effects of three intravenous infusions of infliximab (5 mg/kg) in COPD patients and similarly found no significant improvement in sputum neutrophil levels (132).

Although existing study results do not support the use of infliximab in COPD patients, this does not negate the potential role of TNF-α in the development and progression of the disease. It is important to note that these clinical studies have certain limitations: first, the treatment duration was generally around 6 months, which may be too short for a chronic condition like COPD; second, besides TNF-α, other inflammatory mediators may be sufficient to initiate or sustain COPD symptoms, thereby introducing confounding effects. Therefore, current evidence is insufficient to completely exclude the importance of TNF-α in the pathogenesis of COPD.

#### 3.2.2 Anti-IL-1 therapy

IL-1 is one of the key cytokines involved in the inflammatory response. Elevated levels of IL-1 have been observed in patients with stable COPD and further increase during acute exacerbations, suggesting that high expression of IL-1 may be closely associated with enhanced inflammatory responses in COPD. Therefore, inhibition of IL-1 is considered a potential therapeutic strategy to control the inflammatory process in COPD (133).

Canakinumab is a humanized IgG1 anti-IL-1β monoclonal antibody that highly specifically binds to and neutralizes the biological activity of IL-1β, thereby blocking its signaling and potentially inhibiting COPD-related inflammation (134). A randomized controlled trial (RCT) involving asthma patients has reported that, compared to pre-treatment levels, canakinumab significantly reduced the late asthmatic response (by 28%, P = 0.02). Although this study was small in scale, the results suggest potential clinical benefits (135). On the other hand, research on canakinumab for COPD treatment has also been conducted. A phase II clinical trial evaluated the safety, tolerability, and efficacy of multiple doses of canakinumab (ACZ885) compared with placebo in COPD patients. Unfortunately, the study did not demonstrate a statistically significant improvement in the primary endpoint for canakinumab over placebo (136). Thus, based on current evidence, particularly the negative results from the phase II study, whether canakinumab can be an appropriate treatment option for COPD remains an unresolved question. **Table 2** summarizes the application of drugs targeting different inflammatory pathways in patients with COPD.

**Table 2:** Application of drugs targeting different inflammatory pathways in COPD patients.

Drug	Target	Key clinical trial findings (COPD)	Notes
Inhaled Corticosteroids (113)	Broad-spectrum	Improved lung function, health status, and exacerbation frequency; effective for eosinophilic inflammation	Long-term use associated with hyperglycemia, osteoporosis, etc.
Roflumilast (115)	PDE4 inhibition	Reduced eosinophil counts in sputum and biopsy; improved symptoms	Side effects include nausea, diarrhea, mood changes
Mepolizumab (117, 120)	IL-5	Did not significantly reduce exacerbations; no improvement in lung function or quality of life	Depletes peripheral blood eosinophils
Benralizumab (118, 143)	IL-5R	Phase 2 and 3 trials did not significantly reduce exacerbation rates	Despite eosinophil reduction, clinical outcomes were not improved
Dupilumab (124, 125)	IL-4Rα	Significantly reduced exacerbation rate, improved lung function and quality of life (BOREAS trial)	First biologic to show clear benefit in COPD
Tralokinumab (144)	IL-13	No clinical data in COPD	Approved only for atopic dermatitis
Tezepelumab (127)	TSLP	Phase 2 trial did not significantly reduce exacerbation rate	Further studies needed
Itepekimab (128)	IL-33	Phase 2a trial showed reduced exacerbations and improved lung function (in former smokers)	Progressing to Phase 3 studies
Infliximab (131, 132)	TNF-α	No clinical benefit observed; no improvement in sputum neutrophilia	Treatment duration may have been too short, or other inflammatory mediators may be involved
Canakinumab (136)	IL-1β	Phase 2 trial did not show significant improvement	Positive signals in asthma, but negative results in COPD
Omalizumab (139, 145)	IgE	No COPD clinical trial data	Effective in asthma; IgE elevation is common in COPD, making it a potential target

### 3.3 Immunomodulatory therapy for COPD

It is well established that serum allergen-specific IgE and total IgE play crucial roles in asthma. However, the function of IgE in COPD remains poorly understood. Recent studies have revealed an association between IgE and COPD. A multicenter cohort study (COSYCONET), which investigated the phenotypes and progression of COPD and its comorbidities, demonstrated that elevated total IgE (≥ 0.35 IU/ml) was present in up to 31.2% of COPD patients, with a correlation between total IgE and allergen-specific IgE (r = 0.38, p < 0.001) (137). Another 12-month randomized parallel-group trial (WISDOM) reported similar findings, with 34.2% of COPD patients showing elevated total IgE (138). These studies collectively suggest a potential role for IgE in the pathophysiology of COPD.

Omalizumab, a recombinant humanized anti-IgE monoclonal antibody, has been proven effective in reducing acute exacerbation rates and annual hospitalization rates in asthma treatment (139). Next-generation anti-IgE monoclonal antibodies currently in clinical development may exhibit more potent effects on IgE concentrations. One such monoclonal antibody is ligelizumab (QGE031), which is currently in phase II clinical trials for asthma (NCT02336425). However, the application of anti-IgE monoclonal antibodies in COPD still lacks validation through clinical trials.

### 3.4 Future perspectives

Although extensive research has elucidated specific inflammatory pathways (e.g., aberrant IL-5, IL-13 signaling) and immune dysregulation in COPD patients, the inconsistency between animal model results and clinical efficacy underscores the complexity and heterogeneity of COPD pathogenesis. For instance, studies in murine models of COPD have demonstrated that tobacco smoke-induced inflammation can occur without inflammasome-dependent IL-1β activation (140), a finding that may partly explain the lack of efficacy of targeted biologics in clinical trials for stable COPD. Furthermore, current targeted biologic therapies for COPD have not yet achieved broad success in clinical trials, indicating substantial room for future exploration.

This suggests that future research should focus on precision phenotyping: identifying subgroups of COPD patients with specific driver inflammatory pathways. On this basis, next-generation or more efficient biologics—such as optimized anti-IgE antibodies (e.g., QGE031), antibodies against other novel targets, or bispecific antibodies—may demonstrate potential in these specific subgroups. Concurrently, optimizing clinical trial design—including the selection of more sensitive biomarkers and enrolling patients with specific phenotypes—will be critical for advancing targeted therapies.

## 4 Conclusion

Experimental models of COPD provide a critical platform for systematically elucidating the dynamic changes in immune cells during disease initiation, progression, and acute exacerbations. This review summarizes the infiltration, polarization, functional remodeling, and interactions of key immune cells—such as macrophages, neutrophils, and T lymphocytes—in COPD models, highlighting their central roles in chronic airway inflammation, lung tissue destruction, aberrant repair, and immune dysregulation. Studies demonstrate that immune cells exhibit complex and dynamic evolving patterns in COPD: dysregulated macrophage polarization (M1/M2 imbalance), sustained neutrophil recruitment and dysfunction (e.g., NETosis, delayed apoptosis), amplification of chronic inflammation mediated by T cells (particularly the cytotoxic effects of CD8^+^ T cells, Th1/Th17/Treg imbalance, and formation of tissue-resident memory T cells), along with the collaborative involvement of other immune cells such as fibrocytes and NK cells. These mechanisms collectively shape the unique immune microenvironment characteristic of COPD.

A deeper understanding of these immunological mechanisms not only expands our knowledge of COPD pathological features but also provides critical insights for the development of therapeutic strategies. Clinical evidence indicates that treatment responses in COPD patients are highly dependent on their intrinsic immune-inflammatory phenotypes. For example, biologics targeting eosinophil-associated Th2 inflammation (e.g., anti-IL-5/IL-13 therapies) have shown efficacy in specific subgroups, while dupilumab (anti-IL-4/IL-13) successfully reduced acute exacerbations and improved lung function in Phase III clinical trials, marking a breakthrough in precision immunotherapy. In contrast, most attempts to target neutrophil-associated inflammation (e.g., anti-TNF-α, anti-IL-1β therapies) have failed to achieve desired outcomes, highlighting the complexity of non-Th2 inflammatory pathways and the limitations of current interventions. Meanwhile, conventional drugs (such as PDE4 inhibitors and macrolide antibiotics) and novel strategies (e.g., targeting neutrophil elastase, CXCR2, or pro-resolving mediators) have also demonstrated value in modulating neutrophil function and controlling inflammation. These differential efficacy outcomes not only reflect the high heterogeneity of COPD immunopathology but also reveal the translational gap between animal models and clinical disease.

Future research should build upon existing achievements to precisely characterize the dynamic landscape of immune cells across different stages of COPD, while identifying key biomarkers capable of predicting treatment responses. In this process, it is essential to further explore the interactions between emerging mechanisms—such as immunometabolism and epigenetic regulation—and the dynamic alterations of immune cells. Based on these insights, the development of combined intervention strategies targeting specific immune phenotypes and addressing multiple cells and pathways holds promise for advancing COPD treatment beyond symptomatic control toward delaying or even reversing disease progression, ultimately achieving true precision immunotherapy.
